# Soil Bacterial Assemblage Across a Production Landscape: Agriculture Increases Diversity While Revegetation Recovers Community Composition

**DOI:** 10.1007/s00248-023-02178-x

**Published:** 2023-02-10

**Authors:** A. R. G. Mason, T. R. Cavagnaro, G. R. Guerin, A. J. Lowe

**Affiliations:** 1 School of Biological Sciences, The University of Adelaide, Adelaide, Australia; 2 School of Agriculture, Food & Wine, The University of Adelaide, Adelaide, Australia

**Keywords:** Soil microbial diversity, Impacts of agricultural land use, 16 s rRNA gene sequencing, Above and belowground diversity linkage, Soil physicochemical characteristics

## Abstract

Aboveground ecological impacts associated with agricultural land use change are evident as natural plant communities are replaced with managed production systems. These impacts have been extensively studied, unlike those belowground, which remain poorly understood. Soil bacteria are good candidates to monitor belowground ecological dynamics due to their prevalence within the soil system and ability to survive under harsh and changing conditions. Here, we use soil physicochemical assessment and 16S rRNA gene sequencing to investigate the soil physical and bacterial assemblage changes across a mixed-use agricultural landscape. We assess soil from remnant vegetation (*Eucalyptus* mallee), new and old vineyards, old pasture, and recently revegetated areas. Elevated concentrations of nitrogen (NO_3_^−^) and plant-available (Colwell) phosphorus were identified in the managed vineyard systems, highlighting the impact of agricultural inputs on soil nutrition. Alpha diversity comparison revealed a significant difference between the remnant mallee vegetation and the vineyard systems, with vineyards supporting highest bacterial diversity. Bacterial community composition of recently revegetated areas was similar to remnant vegetation systems, suggesting that bacterial communities can respond quickly to aboveground changes, and that actions taken to restore native plant communities may also act to recover natural microbial communities, with implications for soil and plant health. Findings here suggest that agriculture may disrupt the correlation between above- and belowground diversities by altering the natural processes that otherwise govern this relationship (e.g. disturbance, plant production, diversity of inputs), leading to the promotion of belowground microbial diversity in agricultural systems.

**Supplementary Information:**

The online version contains supplementary material available at 10.1007/s00248-023-02178-x.

## Introduction

The intensification of agriculture that has occurred over the last 100 years, while increasing food production [1], has led to the degradation of many agricultural and natural landscapes [2, 3]. It is perhaps the conversion of natural ecosystems to production systems that is most profound, directly evident by a reduction in aboveground diversity. Production agriculture can also negatively influence soil condition, through depletion of soil organic carbon, acceleration of erosion, reduction of soil fertility, and through acidification and salinisation [2, 4, 5], affecting the productivity and sustainability of aboveground ecosystems [6–9]. Soil degradation leads to reduced agricultural output [10], as well as driving fundamental changes in soil biology [11–14], notably, the balance between component groups of microorganisms, many of which play a pivotal role in broader ecosystem function.

Bacteria perform a variety of functions critical to soil and plant health [15–18]. Bacteria assist in the conversion and uptake of plant available nutrients [19–21], act as phytostimulators promoting plant growth and resilience [22, 23], and biological control agents that protect plants against phytopathogens [24]. Secretions from soil bacteria help form microaggregates by binding soil particles that affect soil structure [25]. Soil microaggregates are increasingly recognised as a characteristic of healthy soil, improving gas exchange, water infiltration, and water holding capacity of the soil [26]. Given the range of functions performed by bacteria, the diversity and composition of their component communities can provide valuable insights into the health and function of associated environments [27–31], including agricultural systems and practices [11, 12, 32–34]. The investigation of soil bacterial communities has been suggested as a way to evaluate the condition of soil and productivity of correlating ecosystems [32, 35–37], with particular relevance to production systems where soil and plant health are intrinsically linked to productivity.

Different land systems and management practices can modify vegetation and soil physicochemical properties, which in turn influence aboveground biodiversity and ecological processes such as nutrient cycling and gas exchange [34, 38–43]. And while there is some evidence that land use practice can modify belowground microbial communities [11–14], it is still unclear how aboveground land systems influence the structure of soil bacterial communities, how much variation exists between and within these communities, and what is driving it [32, 35, 44]. The effect of land use change from natural to managed agriculture on soil bacterial communities is poorly understood, with both positive and negative correlations reported [36, 45–51], while an assumption of above- and belowground diversity linkage still exists. Further investigation across different environments is required to explore these assumptions [52, 53].

Much research has focused on the implications of aboveground land use on soil microbial communities across temperate and mesic biomes [51, 54–57] with considerably fewer studies investigating these interactions in less productive arid systems [36, 58], such as those found throughout much of Australia. Australia’s semi-arid zone occurs through the interior of the continent where average rainfall is between 250 and 500 mm per year. These systems support sclerophyllous vegetation of predominantly low-growing *Eucalyptus* species (commonly termed mallee or mallee scrub), drought-tolerant understory shrubs (e.g. *Acacia* and Chenapod species), and ephemeral grasses and herbs. Here, we investigated the conversion of these systems to production agriculture (vineyards) and the impact of this transition on soil physicochemical characteristics and bacterial community composition. Conversely, we investigate how the restoration of sites with a legacy of agriculture (ex-pastoral land) influences these critical soil components. Given the growing global interest in ecological restoration as a strategy to restore the flow of ecosystem services [6], such investigations are increasingly important in evaluating the success of restorative actions taken. A greater understanding of how the recovery of native plant communities (through active revegetation) influences soil microbial communities can help shed light on the significance of such actions on soil condition.

We hypothesised that managed agricultural systems would be associated with elevated concentrations of key nutrients (nitrate and phosphorus), and that distinct bacterial communities would be associated with different land use systems (i.e. vineyards, remnant mallee vegetation, revegetation, and ex agricultural land). Further, we expected to find a positive correlation between above and belowground diversities, and consequently that the conversion of diverse natural systems (remnant mallee vegetation) to monoculture agriculture (vineyards) would result in a reduction in soil bacterial diversity.

## Materials and method

### Study site

**Fig. 1:**
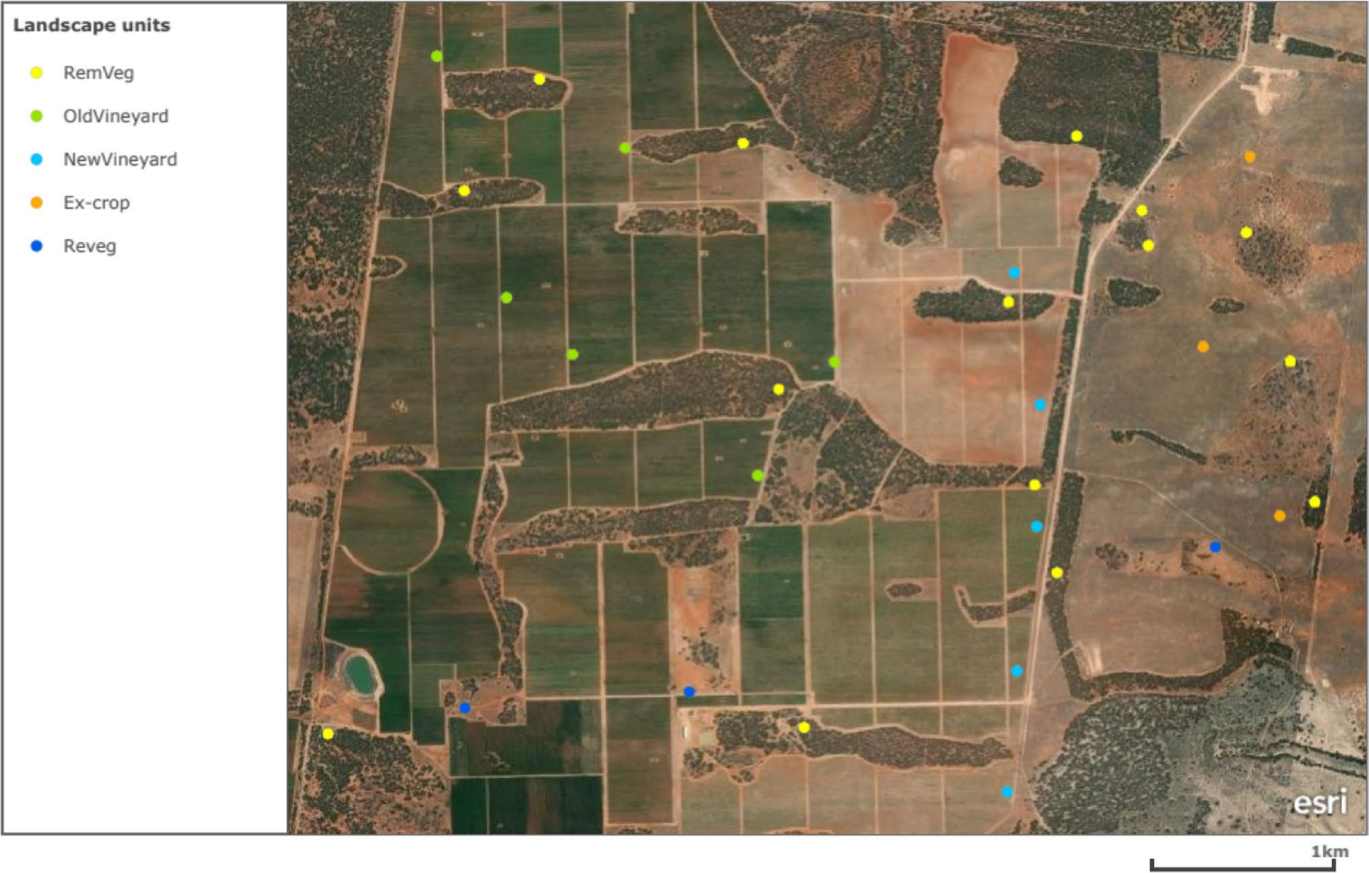
Study system (NSW, Australia) showing the location of the 32 sample replicates owning to the five identified landscape units (ecological systems)

The study site was a mixed-use agriculture production landscape, encompassing agricultural and natural systems (Fig. 1). Located on the River Murray in the New South Wales Murray Darling Wine Region of Australia (34°37′47.8″S, 143°00′56.2″E), the site consisted of two commercial agricultural operations: wine vineyards and dried fruit vineyards. The region is highly productive producing high-value crops such as grapes, citrus, olives, nuts, stone fruit, cereal crops, and livestock. The area was classified as semi-arid with most of the annual average rainfall of ~ 300 mm falling during the Austral winter (i.e. June–August) [59]. Soil, aspect, and elevation were consistent across the site, with soils classified as calcarosols or mallee loam, ranging from brown to red-brown loamy sand, sandy loam, or loam [60].

Much of the site was dominated by irrigated vineyards (roughly 50%), which has replaced the remnant native *Eucalyptus* mallee that would have occurred across most of the site and of the region prior to its conversion to agriculture (Fig. 1). The management practices of both vineyard systems were consistent, both being applied with two microbial inoculants (Supplementary 7). Along with the active vineyard operations and remaining remnant mallee vegetation, an ex-pastoral/cropping section existed along the north-eastern boarder of the site (~ 420 ha). This section was abandoned for agricultural use within the last 5 years (assessed as unsuitable for irrigated agriculture) although still possesses a legacy of past cropping and pastoralism via a system dominated by wheat, and mixed native and introduced grasslands.

### Ecological systems

**Fig. 2:**
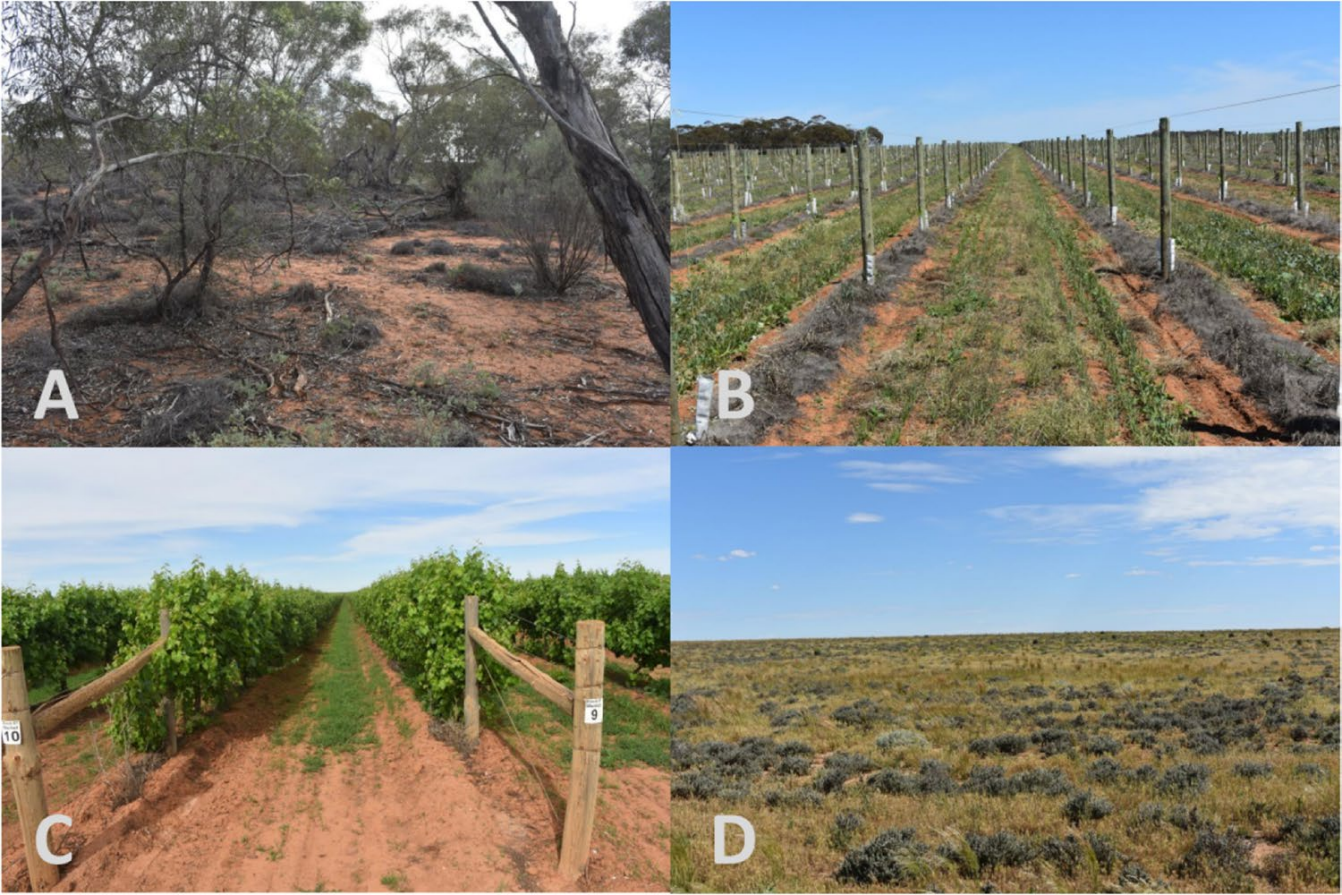
Sample images of landscape unit replicates; **A** remnant mallee vegetation (RemVeg), **B** new vineyard (NewVineyard), **C** established vineyard (OldVineyard), **D** pastoral land abandoned for agricultural use (ExCrop)

Five distinct land use/ecological systems were identified across the study site (landscape units, hereafter) (Figs. 1 and 2). Remnant mallee vegetation of mixed *Eucalyptus* (*E*. *gracilis*, *E. brachycalyx*, *E. leptophylla*, *E. incrassata*) (RemVeg, hereafter) was identified and used as the natural reference system in which the impact of land use change could be measured against. Three agricultural landscape units were identified: established vineyards (OldVineyard, hereafter) consisting of grape vines over 10 years old, new vineyards (NewVineyard, hereafter) consisting of grape vines under 2 years old, and a grassland section (old pasture) that had been abandoned for agricultural use (Excrop, hereafter). Finally, a native revegetation landscape unit (Reveg, hereafter) was identified, consisting of three plantings undertaken within 2 years of sampling (2019–2020); one seedling planted site, and two direct seeded revegetation sites which comprised a seed mix of approximately 15 local native plant species that had not yet emerged at time of sampling.

Replicates were identified for each of the five landscape units, totaling 32 individual sampling sites, broken down as follows; 15 replicates of the RemVeg landscape unit, six replicates of the OldVineyard landscape unit, five replicates of the NewVineyard landscape unit, three replicates of the Reveg landscape unit, and three replicates of the Excrop landscape unit (Fig. 1). The sampling design was developed with consideration to soil and landscape variation (aspect and elevation). Replicates were chosen based on their location across the study site, where possible landscape units were identified and sampled that were in close vicinity to one another and in consistent soil types, allowing for comparative analysis between each.

### Soil sampling

Soils were sampled following the Biomes of Australian Soil Environments (BASE) project protocol [61], in December 2020 (i.e. Austral Summer). Briefly, one soil sample was taken from each of the landscape unit replicates (*n* = 32), which comprised three pooled sub-samples taken from a 30 m radius within the replicate. Soil was collected from the bulk soil surface horizon (0–10 cm depth), a portion (approx. 50 g) of which was stored in a sterile 50-mL tube to be used for DNA extraction, and another larger portion (~ 300 g) stored in a ziplock bag for physicochemical analysis. The top litter layer was carefully removed and a scoop taken to required depth (10 cm), with the three sub-samples thoroughly mixed prior to portioning. Samples to undergo physicochemical analysis were air dried in bag and stored at room temperature, while tubes where immediately stored at − 8 °C until microbial analysis was performed.

Additional metadata were also collected at each of the landscape unit replicates, consisting of photos of each sub-sampling location, GPS coordinates, and notes on vegetation community variables including a plant species list. Plant species lists were compiled via visual inventories during a 5-min search of the immediate area surrounding the sampling location (30 m soil sampling radius).

### Soil analysis

Soil physicochemical analysis was undertaken by the Australian Precision Ag Laboratory (APAL, Adelaide, Australia). Specifically, ammonium (NH_4_^+^), nitrate (NO_3_^−^), plant-available (Colwell) phosphorus, potassium, sodium, magnesium, calcium, organic carbon, soil pH (CaCl_2_), and soil texture were quantified. The Colwell phosphorus test employed provides a measure of plant-available phosphorus, that being the bicarbonate-extractable phosphorus. The Colwell phosphorus method is considered to estimate phosphorus quantity and is the most common soil phosphorus test used in Australia [62]. Organic carbon was determined using the Walkley and Black wet oxidation method, providing an approximation of total soil organic carbon by measuring the readily oxidisable/decomposable carbon which is considered to account for roughly 80% of the total soil organic carbon pool [63]. DNA extraction and sequencing were undertaken by the Australian Genome Research Facility (AGRF, Adelaide, Australia) using the ‘DNeasy PowerSoil Pro Kit’ from Qiagen [64]. Briefly, soil samples were added to a bead beating tube for rapid and thorough homogenisation, cell lysis occurred by mechanical and chemical methods, total genomic DNA was captured on a silica membrane in a spin column format, and DNA was washed and eluted from the membrane ready for downstream analysis [64].

Bacterial 16S ribosomal rRNA was PCR amplified for each replicate using the forward 27f (AGAGTTTGATCMTGGCTCAG) and reverse 519r (GWATTACCGCGGCKGCTG) primers. Sequence data was analysed using the QIIME 2 (2019.7) platform [65]. The demultiplexed raw reads were primer trimmed using the cutadapt plugin, with a length cut-off of 240 bp for the forward primer (default –error-rate 0.1 –times 1 –overlap 3). DADA2 with default setting (–p-max-e 2, –p-chimera-method consensus) was used to denoise, dereplicate, and filter chimeras [66]. Taxonomy was assigned to amplicon sequence variants (ASVs) using the q2 feature classifier [67]. Sequences from the Greengenes databases (v13.8) were trimmed for the targeted regions (V1–V3) and used as a training dataset for the classifier resulting in an absolute abundance ASV table to be used in downstream analysis.

### Statistical analysis

The vegetation data (plant species list) was used to determine the mean plant functional diversity associated with each of the landscape units (Supplementary 5 and 6). Observed species were categorised into one of five functional groups; perennial herbaceous groundcover (0–30 cm height, grasses and forbes); annual herbaceous groundcover (0–30 cm height, grasses and forbes); small shrub (< 1 m height, woody perennial); medium/large shrub (> 1 m height, woody perennial); and tree (woody plants with trunk and canopy over 3 m height). The observed plant functional groups were summed for each replicate, providing a plant functional diversity score for each sample replicate from which a mean plant function diversity score could be derived for each landscape unit.

The majority of statistical analysis was undertaken using R software (v4.03) [67, 68], employing the microbiome data analysis framework of the *phyloseq* package (v1.32.1) [69]. Both rarefied and non-rarefied data were analysed dependent on input and standardisation requirements of particular analysis. Firstly, rare sequence variants were removed (< 10 sequence reads) from the ASV table, using the ‘prune_taxa’ function of the *phyloseq* package. A linear model (LM) was used to identify significant relationships between soil variables and landscape units using the ‘lm’ function of the *phyloseq* package. To investigate differences in community composition between landscape units, ordination of ASV beta diversity was calculated with the ‘ordinate’ function in *phyloseq* using unrarefied data. Constrained analysis of principal coordinates (CAP) was performed on the Bray–Curtis dissimilarity matrix constrained by soil variables: organic carbon, nitrate, phosphorus, sodium, pH, calcium, magnesium, ammonium, and by plant functional diversity. Potassium was removed from ordination, as it was highly correlated with other variables (significance cut-off of > 0.7 or <  − 0.7, Pearson’s product-moment correlation). Constraining variable significance was assessed non-parametrically via 999 permutations. The ‘betadisper’ function was used to test for homogeneity of group dispersions. A PERMANOVA (999 iterations) was run with the ‘pairwise_adonis2’ function of the pairwise.adonis package to test the significance of community compositional variation between landscape units [70].

To investigate diversity of landscape units, alpha diversity was calculated at ASV level using observed richness and Shannon and Simpson diversity indices, performed using the ‘estimate_richness’ function in the phyloseq package. Prior to alpha diversity calculations, ASV level data was rarefied to using the phyloseq packages ‘rarefy_even_depth’ function, and Shannon and Simpson index values were transformed to effective number of ASVs. A negative binomial generalised linear model (GLM) was used to test for differences in alpha diversity between landscape units, followed by ‘goodness of fit’ analysis using chi-squared distribution ‘pchisq’ function in the phyloseq package. A type II Wald chi2 test was run with the ‘ANOVA’ function of the car package to test main effects of the GLM’s (v3.0–10) [71]. Pairwise comparisons using Holm-Bonferroni *P*-adjustment were then made between landscape units using ‘pairwise’ function in the phyloseq package. A correlation matrix (Pearson product-moment) was used to identify significant relationships between soil and ecological (plant functional diversity) variables and diversity metrics, and to determine any correlating variables. Bacterial community composition was further investigated via a relative abundance stack plot, created by converting the rarefied family abundances to percentages. Rare families (< 2% of total rarefied sequences) were pooled into a single group named ‘pooled (< 2% relative abundance)’.

## Results

### Aboveground diversity

Remnant mallee vegetation (RemVeg) had the highest plant species richness and plant functional diversity, possessing all five functional groups across replicates (Table 1; Supplementary 5 and 6). Four plant functional groups were observed across replicates of the revegetation system (Reveg), which was also found to contain some large established and recruiting native vegetation. Seedlings of planted species were not included in the Reveg landscape unit species list, as they were not yet established. The managed vineyard systems (OldVineyard and NewVineyard) were found to have low plant functional diversity, typically consisting of a medium shrub layer (*Vitis* sp.) and an annual grassy groundcover. Likewise, the ex-pasture systems (ExCrop) predominantly consisted of two plant functional groups (perennial and annual herbaceous groundcover).

**Table 1:** Plant species richness and functional diversity results. Average plant functional diversity figures represent the mean number of plant function groups present across landscape unit replicates; 5 indicating all function groups present, 1 indicating one functional group present (Supplementary 5)

Landscape unit	Plant species richness	Mean plant functional diversity
RemVeg	30	3.6
Reveg	9	3
ExCrop	8	2.7
OldVineyard	3	2
NewVineyard	3	1.8

### Soil physicochemical properties

Soil texture was consistent across the study site (Supplementary 1). Replicates of the remnant mallee vegetation landscape unit (RemVeg) ranged from sandy loam to silty loam. Similarly, both the vineyard landscape units (OldVineyard and NewVineyard), the revegetation (Reveg), and the ex-cropping landscape units (ExCrop) were identified as either silty loam, sandy loam, or loam.

**Fig. 3:**
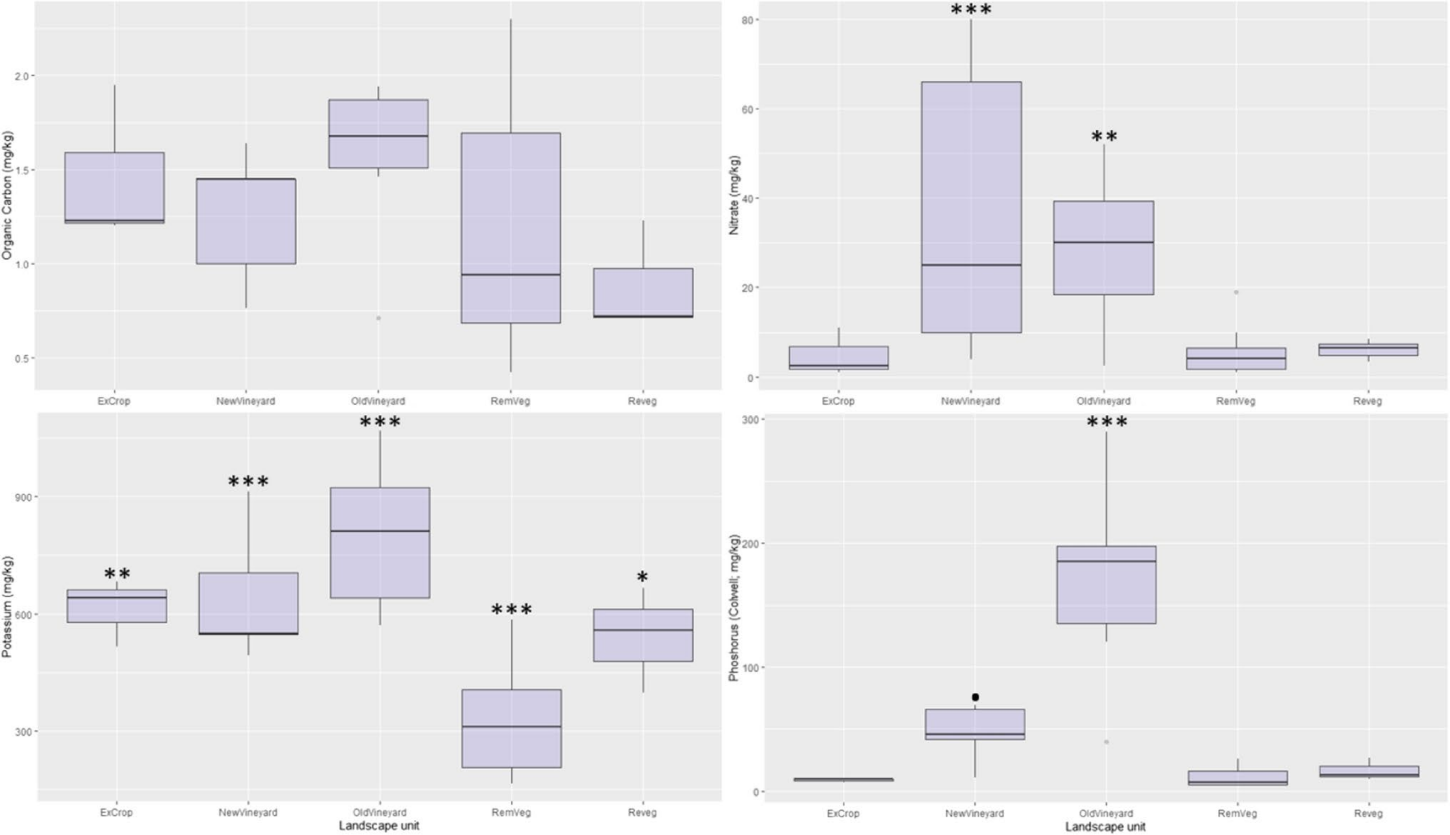
Boxplot panel displaying organic carbon (mg/kg), nitrate (NO3.^−^; mg/kg), potassium (mg/kg) and phosphorus (Colwell; mg/kg) concentrations across landscape units, with linear model (soil variable by landscape unit) significance codes: ‘.’ *P* < 0.10; ‘*’ *P* < 0.05; ‘**’ *P* < 0.01; ‘***’ *P* < 0.001 (Holm-Bonferroni *P*-adjustment)

**Fig. 4:**
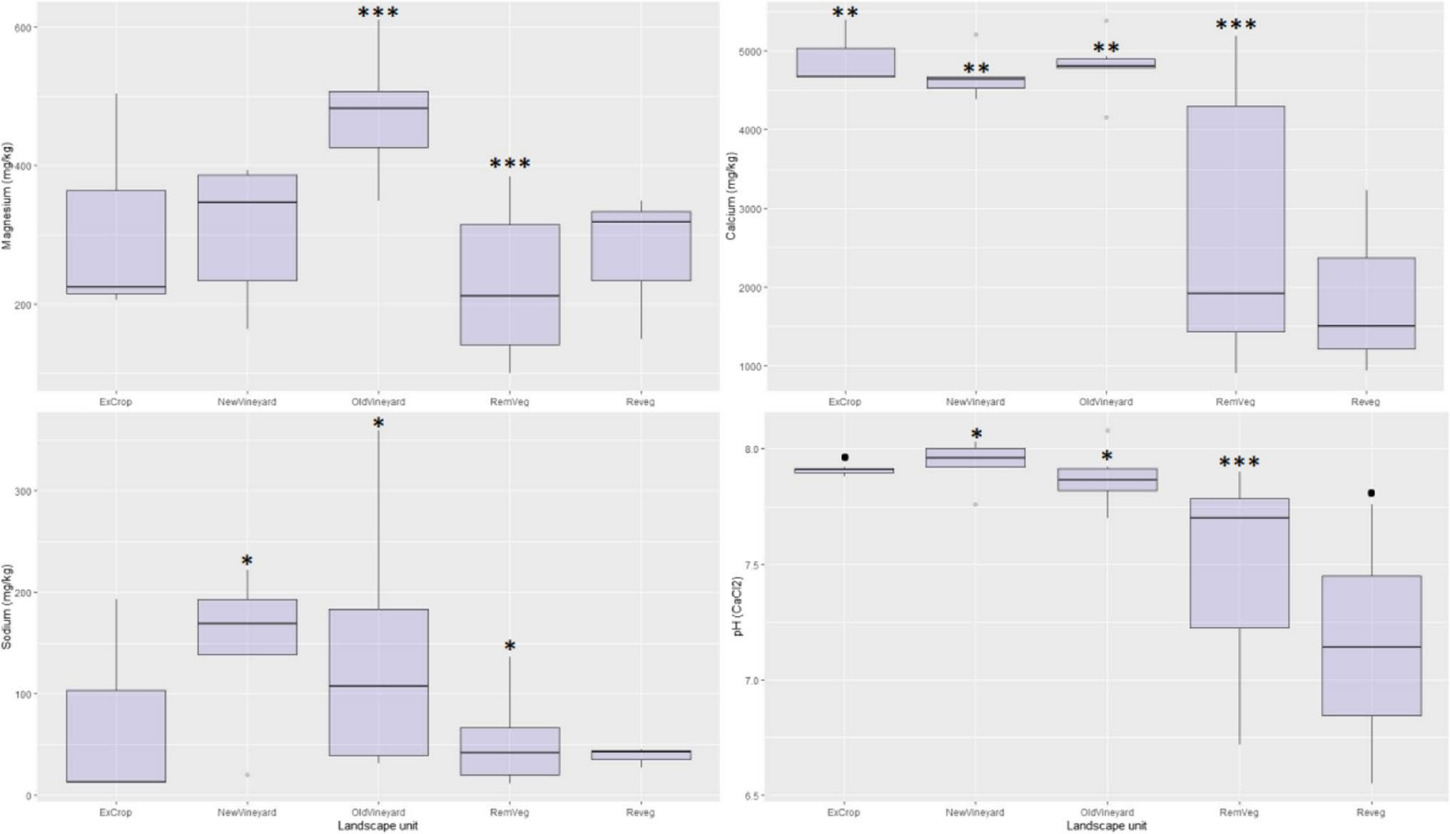
Boxplot panel displaying magnesium (mg/kg), calcium (mg/kg), sodium (mg/kg), and pH (CaCl_2_) concentrations across landscape units, with linear model (soil variable by landscape unit) significance codes: ‘.’ *P* < 0.10; ‘*’ *P* < 0.05; ‘**’ *P* < 0.01; ‘***’ *P* < 0.001 (Holm-Bonferroni *P*-adjustment)

Linear models (soil variable against landscape units) revealed a number of statistically significant correlations between physicochemical variables and landscape units, including a number of key nutrients. Nitrate (NO_3_^−^) was highest in both vineyard systems (NewVineyard, *p* < 0.001; OldVineyard, *p* = 0.004), plant-available (Colwell) phosphorus was elevated in the OldVineyard landscape unit (*p* < 0.001), while potassium was significantly lower in the RemVeg landscape unit (Fig. 3). Magnesium was elevated in the OldVineyard (*p* < 0.00) landscape units, calcium was elevated in the ExCrop (*p* = 0.007), NewVineyard (*p* = 0.003) and OldVineyard (*p* = 0.001) landscape units (Fig. 4), and sodium was elevated in the two vineyard systems (new, *p* = 0.015; old, *p* = 0.020).

### Bacterial community composition

**Fig. 5:**
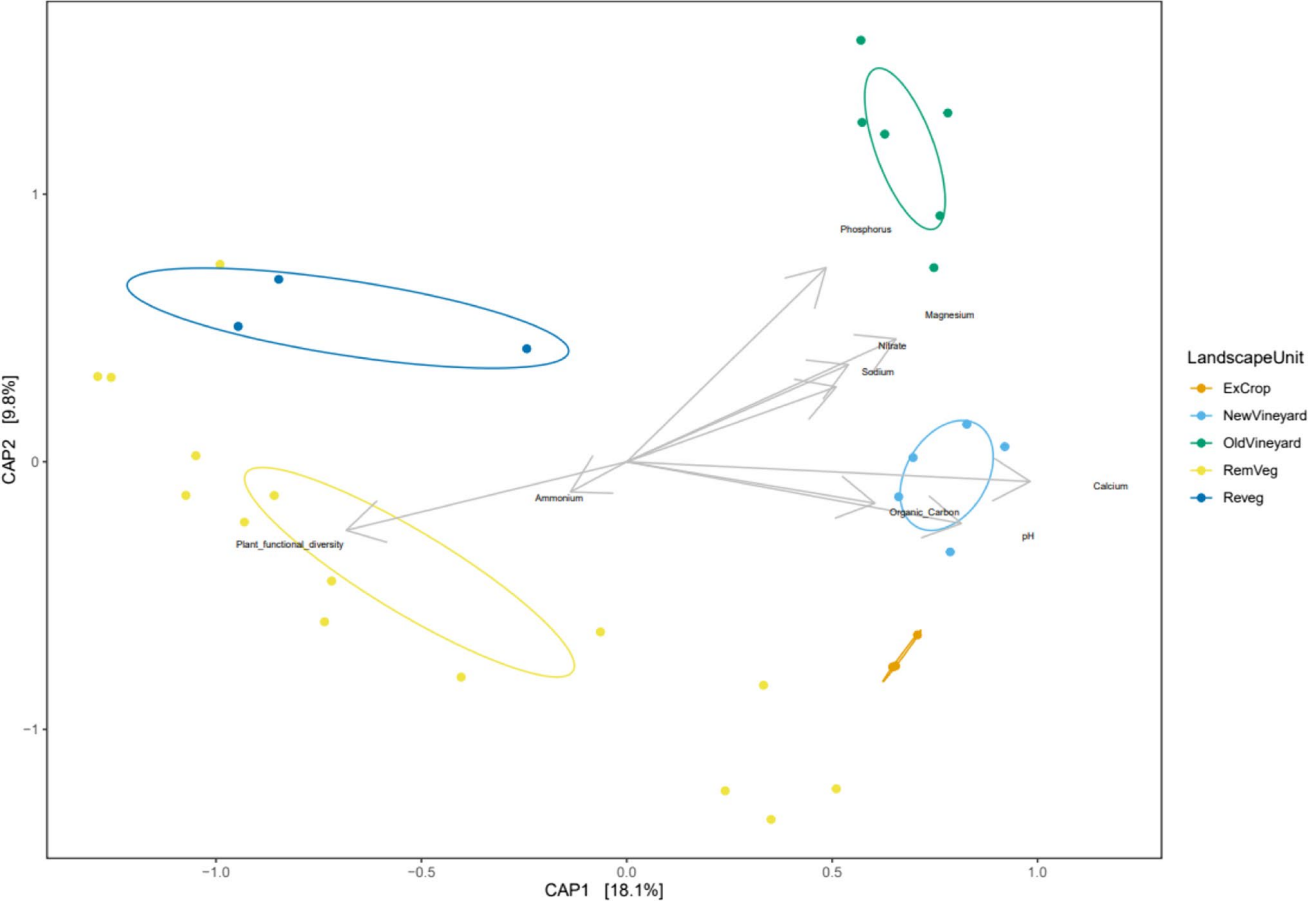
First two axes of a constrained analysis of principal coordinates (CAP) using Bray–Curtis distance constrained by soil physicochemical and ecological (plant functional diversity) variables, which explained 48% of variance across all axes. Axes 1–2 (depicted) explain 18.1% (CAP1) and 9.8% (CAP2) of total variance, respectively. 95% confidence ellipses were applied post-hoc to landscape units

Landscape units were found to be associated with distinct bacterial communities (Fig. 5), with a significant PERMANOVA test on the dissimilarity matrix (Bray–Curtis, *F* = 2.86, *p* < 0.001). Pairwise community analysis revealed a significant difference in community composition between all but one of the 10 landscape unit pairwise comparisons, that being the ExCrop and Reveg landscape units (Supplementary 4). Constrained ordination (CAP) indicated a clear shift in community composition from the more natural landscape units (RemVeg and Reveg) to the highly modified systems (ExCrop, OldVineyard, and NewVineyard) (Fig. 5). Constraining gradients of soil physicochemistry and plant functional diversity explained 48% of variance in community composition, with organic carbon, nitrate, phosphorus, pH, and calcium found to be significant (Table 2). Beta dispersion test was significant (*F* = 17.839, *p* < 0.001), indicating that landscape units had variable species turnover among replicates.

**Table 2:** Permutation test for capscale under reduced model, 999 permutations. (formula = OTU ~ organic carbon + nitrate + phosphorus + sodium + pH + calcium + ammonium + magnesium + plant functional diversity). Significance codes: 0 ‘***’ 0.001 ‘**’ 0.01 ‘*’ 0.05 ‘.’ 0.1 ‘’ 1

Variable	DF	SumOfSqs	*F*	*P*	Sig
Organic carbon	1	0.938	3.910	0.001	***
Nitrate	1	0.614	2.558	0.002	**
Phosphorus	1	0.749	3.121	0.001	***
Sodium	1	0.255	1.062	0.325	
pH	1	0.829	3.454	0.001	***
Calcium	1	0.676	2.816	0.001	***
Ammonium	1	0.231	0.962	0.496	
Plant functional diversity	1	0.292	1.218	0.196	
Magnesium	1	0.340	1.415	0.079	
Residual	22	5.279			

The more natural predominantly unmanaged native vegetation land systems (RemVeg and Reveg) separate out from the highly modified managed systems (OldVineyard, NewVineyard, and ExCrop) along the primary *x* axis (CAP1, 18.1% of variation explained) (Fig. 5). This partitioning appears to be strongly influenced by plant functional diversity and agricultural inputs, evident by vectors plant functional diversity, nitrate, phosphorus, calcium, and magnesium with soil pH also an influential variable affecting group partitioning along the *x* axis (CAP1). Bacterial community shift is also apparent within the highly modified and managed systems along the *y* axis (CAP2, 9.8% of variation explained), with the more natural ExCrop landscape unit (in comparison to the vineyard systems) clearly removed from the established vineyard system (OldVineyard), with the newly established vineyard system (NewVineyard) sitting between the two (Fig. 5).

### Bacterial alpha diversity

**Fig. 6:**
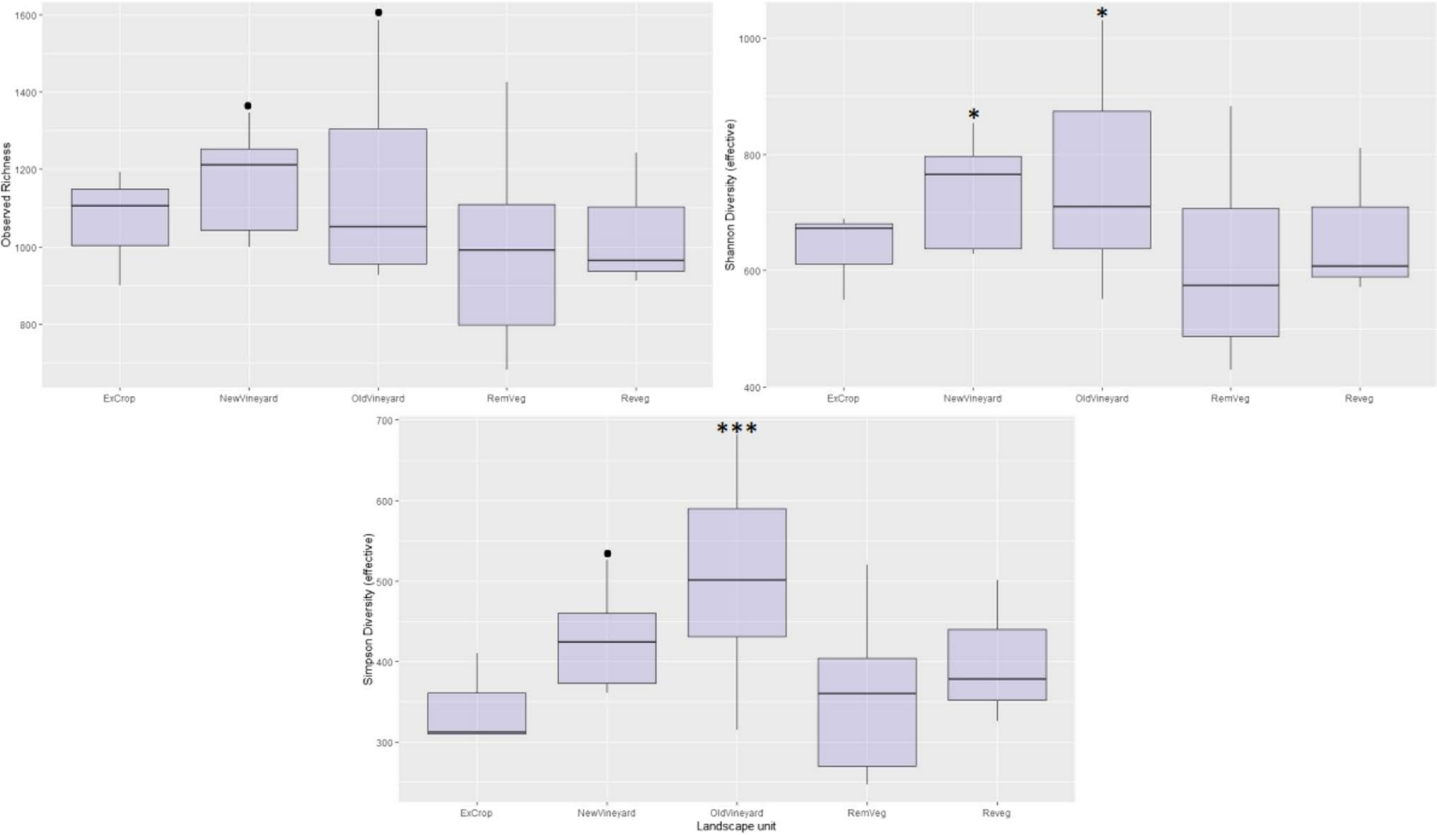
Boxplot of alpha diversity results across landscape units, displaying observed richness, Shannon (effective species), and Simpson (effective species) diversity metrics, with negative binomial GLM model (diversity metric by landscape unit) significance codes: ‘.’ *P* < 0.10; ‘*’ *P* < 0.05; ‘**’ *P* < 0.01; ‘***’ *P* < 0.001 (Holm-Bonferroni *P*-adjustment)

Soil bacterial diversity was compared between landscape units at ASV level. Of the three diversity metrics calculated (observed richness, effective Shannon, and effective Simpson), Simpson diversity was found to be significantly different among landscape units, as determined by GLM (Simpson, chi^2^ = 0.225, *p* = 0.005; Shannon, chi^2^ = 0.225, *p* = 0.078) (Supplementary 2). The managed vineyard systems returned the highest bacterial diversity across all measured metrics, with both these systems returning significant Shannon diversity (NewVineyard, *Z* = 2.037, *p* = 0.042, OldVineyard, *Z* = 2.483, *p* = 0.013), while the OldVineyard returned significant Simpson diversity (*Z* = 3.496, *p* = 0.0004) (Fig. 6, Supplementary 2).

Alpha diversity pairwise comparisons revealed a significant difference (Simpson diversity) between the RemVeg and OldVineyard landscape units (*Z* =  − 3.496, *p* = 0.004) (Supplementary 3). No significant correlations (cut-off of 0.7, Pearson’s product-moment correlation) were found between any of the soil physicochemical or ecological (plant functional diversity) variables measured and any of the calculated diversity metrics (observed richness, Shannon, Simpson) (Table 3). Several significant correlations were found between soil physicochemical variables (potassium/calcium, *r* =  + 0.722; potassium/phosphorus, *r* =  + 0.747; potassium/plant functional diversity, *r* = 0.675).

**Table 3:** Results from correlation matrix (Pearson product-moment), soil variables, and diversity metrics. Values of > 0.7 or <  − 0.7 indicate significant correlation

Variable	Observed	Shannon	Simpson
Ammonium	− 0.232	− 0.233	** − **0.218
Calcium	0.031	− 0.004	**0.011**
Magnesium	− 0.118	− 0.079	**0.010**
Nitrate (NO3^−^)	− 0.011	0.055	**0.113**
Organic carbon	− 0.330	− 0.370	** − **0.313
pH (CaCl_2_)	0.080	0.034	**0.046**
Phosphorus (Cowell)	0.038	0.099	**0.225**
Potassium	0.114	0.115	**0.112**
Sodium	− 0.207	− 0.151	** − **0.089
Plant functional diversity	** − **0.136	** − **0.128	** − **0.097

### Taxa analysis

Bacterial taxa analysis was undertaken at the family taxonomic level, as it was expected that more specific and precise functional information could be sought because it was thought that similar functional groups (e.g. decomposers, parasites, mutualists) would be represented. Analysis revealed that rare taxa (< 2% relative abundance) dominate the system (34% relative abundance), with only 10 of the 210 families identified across the site found to be abundant (> 2% relative abundance), these being *Rubrobacteraceae* (17.4% relative abundance), *Bacillaceae* (9.2% relative abundance), *Bradyrhizobiaceae* (7.2% relative abundance), *Pseudonocardiaceae* (3.6% relative abundance), *Micrococcaceae* (3.1% relative abundance), *Geodermatophilaceae* (2.7% relative abundance), *Rhodospirillaceae* (5.1% relative abundance), *Sphingomonadaceae* (3.4% relative abundance), *Sinobacteraceae* (2.8% relative abundance), and *Hyphomicrobiaceae* (2.7% relative abundance).

**Fig. 7:**
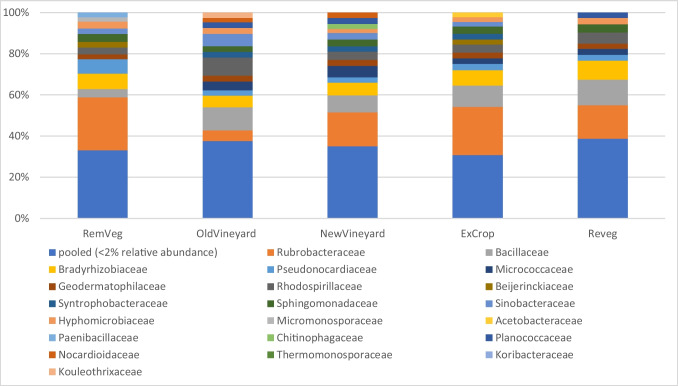
Bacterial family stack plot (mean relative abundance). Displaying landscape unit bacterial community composition at family level

The relative abundance of bacterial families within landscape units was further investigated, similarly finding that bacterial communities were dominated by rare taxa (< 2% relative abundance), ranging from 38% of community composition in the Reveg landscape unit to 30% in the ExCrop landscape unit (Fig. 7, Supplementary 8). Interestingly, the Rubrobacteraceae family was found to be the dominant taxa in all landscape unit communities except the established vineyard system (OldVineyard), where the *Bacillaceae* family was found to be most relatively abundant (Fig. 7, Supplementary 8).

## Discussion

Bacterial community composition and soil physicochemical characteristics of different land systems (landscape units) were investigated across a semi-arid production landscape to explore the impact of land use on soil bacterial communities. As hypothesised, the landscape units differed in their soil physicochemical characteristics. This was linked to a shift in the soil microbiome, such that distinct bacterial communities were associated with land use systems. Interestingly, highest bacterial diversity was observed in the managed vineyard system, highlighting that aboveground diversity does not necessarily correlate with belowground diversity. The restoration of native plant communities appears to be acting to recover native bacterial communities, suggesting that such actions have the capacity to not only influence aboveground species composition but also belowground bacterial community assemblage.

### Managed vineyard systems associated with elevated levels of key nutrients

Elevated concentrations of nitrate (NO_3_^−^) and plant-available (Colwell) phosphorus were identified in the managed vineyard systems (OldVineyard and NewVineyard). The higher concentrations of these nutrients were not surprising, given the addition of soil microbial inoculants containing proportions of these nutrients (product A, nitrogen = 2.66% w/v, phosphorus = 1.2% w/v, potassium 0.25% w/v; product B, phosphorus = 2.09%), and the addition of other fertilisers that would also likely contain these nutrients.

This result serves to highlight the physiochemical changes in agricultural systems (mallee vegetation to vineyard agriculture) in relation to soil nutrition. Although increased concentrations of common agricultural inputs such as nitrogen and phosphorus could be viewed as positive in the context of agricultural productivity, the long-term sustainability of the system could be questioned given the well-recognised negative impacts associated with fertiliser use [2, 72].

### Land systems/practices drive distinct bacterial communities

Our results indicate that bacterial community composition is strongly associated with land use, based on pairwise comparisons and constrained ordination (CAP) analysis of bacterial community composition (Fig. 5, Supplementary 4). Only one of the pairwise comparisons was not significant (ExCrop – Reveg). This is likely due to the fact that the revegetation systems have only recently (within the last 2 years) been converted from pastoral land (ExCrop), and as such the associated bacterial community still resembles the community associated with the ExCrop landscape units. The observed separation of natural systems (RemVeg and Reveg) from the modified agricultural systems (ExCrop, OldVineyard, and NewVineyard) in the CAP analysis indicates that agricultural land use change has modified bacterial community composition across the study system. The observed partition of natural and agricultural communities appears to be strongly correlated with, and potentially driven by, plant functional diversity and management practice.

Elevated concentrations of key nutrients in the vineyard systems may be the result of microbial inoculants and additional fertiliser inputs, indicating that these practices, and the associated change in soil nutrients, have influenced community composition, evident by statistically significant nitrate and phosphorus-constraining variables in CAP analysis (Fig. 5). Although not found to correlate with bacterial diversity (Table 3), soil pH also appears to influence community composition, in line with other studies returning similar results [73]. Constrained ordination (CAP) also revealed that plant functional diversity influenced community composition in the opposite direction to key nutrient vectors (nitrate and plant-available (Colwell) phosphorus) in the ordination space (Fig. 5). As expected, the conversion of remnant mallee vegetation to vineyard agriculture was found to reduce plant functional diversity, suggesting that land use change is also a key factor influencing bacterial community composition.

The Rubrobacteraceae family was the most abundant family across the study site (~ 17.4% relative abundance) and was most abundant in all landscape units except the established vineyards (OldVineyard). Recognised as one of the most radiation-resistant organisms [74], and halotolerant and desiccation tolerant [75], the Rubrobacteraceae has a selective advantage in extreme environments, including arid soils [74, 76], permafrost [77], and saline environments [78]. The greater relative abundance of Rubrobacteraceae across the study system is likely a legacy of the semi-arid soils that much of the site would have consisted of before its conversion to irrigated production agriculture. The reduced abundance of this family in vineyard systems highlights the ability of land use to alter the abundance of specific taxa. Indeed, revegetation of old pasture sites (Reveg landscape unit) has acted to shift the bacterial community back towards a reference state (Fig. 5)—community associated with remnant mallee vegetation (RemVeg). This finding suggests that the restoration of aboveground ecosystems can act to restore belowground bacterial communities, as reported in other studies [79], with potential implications for soil and wider ecosystem health. For instance, native microbial communities likely harbor a greater proportion of species possessing advantageous traits to local environmental conditions, providing a pool of well adapted (potentially plant beneficial) species that may disperse to adjacent production systems, such as vineyards.

To explore the efficacy of management practices employed to improve soil condition (inoculation), inoculated groups were investigated (where possible). The inoculated bacterial family *Pseudomonadaceae* was not found to be abundant (> 2% relative abundance) in any of the landscape units (Supplementary 8), including the landscape units in which it was applied (OldVineyard and NewVineyard), indicating that the addition of inoculants has not influenced the abundance of this family. No conclusion could be drawn regarding the efficacy of applied inoculants to proliferate members of the Actinomycetes group, as no information could be sourced regarding which specific taxon’s (e.g. species, genera, families) the inoculants contained.

### Conversion of remnant mallee vegetation to vineyard agriculture increases bacterial diversity

Observed bacterial richness at amplicon sequence variant level was not significant among the seven landscape units assessed, while both effective Simpson and Shannon diversity metrics (which account for abundance and evenness) were found to be statistically significant (Supplementary 2). Shannon and Simpson diversity metrics are widely recommended and commonly used when analysing microbial diversity and have been shown to reduce the bias (richness over evenness) often associated with other diversity metrics [80, 81].

Results revealed that the managed vineyard landscape units had highest soil bacterial diversity, with both the established and new vineyard systems (NewVineyard and OldVineyard) returning statistically significant diversity results. This result was not in line with our expectations or with other studies that suggest a positive correlation between plant diversity/complexity and bacterial diversity [12, 51, 53, 81]. The conversion of the more diverse remnant mallee vegetation to monoculture agriculture has, in fact, increased belowground bacterial diversity, suggesting that agriculturally driven land use change has resulted in a decoupling of above- and belowground diversities.

Although a positive relationship between above- and belowground diversities has been observed in other studies [53], and could be considered a broadly accepted principle [82], we suggest that agriculture can act to disrupt this relationship via major modification of the natural soil system; modification occurring through the removal and replacement of natural plant communities (and their associated inputs), and ongoing management practices associated with production systems, such as the planting of crops, chemical/fertiliser application, and soil tillage. In this regard, we propose that agricultural land use (and associated practices) may be a stronger driver of soil bacterial diversity than aboveground plant diversity. In their analysis of experimental grasslands, Zak et al. (2003) found that plant diversity increased the biomass and composition of soil microbial communities, but attributed this to the increase in plant production associated with greater species diversity, rather than to plant diversity per se [83]. This goes some way to explaining findings here, given that plant production would likely be greatest in the agricultural systems (due to management practices such as fertiliser and water inputs) where bacterial diversity was also found to be highest (New and OldVineyards), adding support for our suggestion that agriculture fundamentally disrupts the natural processes that otherwise govern above- and belowground diversity linkage, such as plant production. Further support for this can be found in a global meta-analysis of more than 84 studies. Liu et al. (2020) found that microbial richness showed a moderate but positive correlation with plant diversity, and likewise suggested that plant communities with higher diversity may promote more diverse microbial communities through greater diversity of inputs (chemical, i.e. root exudates and physical, i.e. litter) and higher productivity, leading to increased niche space [53].

Another factor likely impacting diversity, and typical of agricultural systems, is disturbance. The intermediate disturbance hypothesis (where the diversity of competing species will be maximised at intermediate frequencies and/or intensities of disturbance or environmental change) [84] partly explains the diversity results found here. In stable-state environments, fewer well-adapted taxa outcompete and dominate those less adapted, reducing diversity. Conversely, intermittently disturbed environments can result in the persistence of a greater number of taxa due to increased environmental heterogeneity or habitats (niche space) in which different taxa are suitably adapted, and can exploit [16, 36]. Although it is recognised that the study here is not appropriately designed to test the IDH, results do point to disturbance as a significant factor driving bacterial diversity.

Land use change (from remnant mallee vegetation to managed vineyards) and associated management practices employed (e.g. cover cropping, chemical/fertiliser application, tillage) could be viewed as having a positive effect on soil health, given that there is some evidence to suggest that soil microbial diversity confers stability to stress and protection against soil-borne disease [84]. However, while microbial diversity is a valuable tool in evaluating change in soil condition, it is acknowledged that increased diversity does not necessarily indicate positive change. For instance, a bacterial community may have higher diversity than another while also consisting a greater proportion of parasitic taxa, potentially indicating poor soil condition (in the context of plant productivity). Thus, taxonomic community shifts should also be considered in evaluating the impacts of aboveground land use change, as this variable may be of greater significance to soil–plant systems than diversity per se. It is also recognised that increased bacterial diversity and soil fertility associated with the managed vineyard systems are likely the results of other inputs, such as microbial amendments, fertilizer, and water. Interestingly, nitrate and plant-available (Colwell) phosphorus were found to be elevated in both vineyard systems (Fig. 3), while no significant correlation was identified between these nutrients and bacterial diversity (Table 3). It could be assumed that the amendments (hydrolysed molasses, amino acids, fulvic acid, seaweed, and liquid fish) and/or the living bacteria within the inoculants are driving diversity in the inoculated systems (New and OldVineyards) [85]. However, this assumption lacks the appropriate statistical evidence to be validated here, and further work would be required to assess the impact of these soil amendments on bacterial diversity.

## Conclusion

A reduction in aboveground plant diversity is inevitable when natural systems are converted to large-scale production monocultures, as was found here. It is also broadly assumed that this aboveground change results in a reduction in belowground diversity (above- and belowground diversity linkage). Our results stand in contrast to this assumption with the finding that agricultural systems (with reduced aboveground diversity) have increased soil bacterial diversity. We highlight that restoration of native plant communities can act to rapidly recover natural soil bacterial communities, which in turn could improve soil and plant health. However, the impact of shifts in bacterial community composition associated with land use systems detected here is not fully understood without a greater understanding of the functional significance of the key bacterial groups identified. Such shifts should be considered in future studies seeking to further our understanding of land use impacts on soil and plant health.

## Supplementary Information

Below is the link to the electronic supplementary material.

Supplementary file1 (DOCX 52 KB)

## Data Availability

The datasets generated during the current study are available in the Figshare repository, https://doi.org/10.6084/m9.figshare.19289450
