# Supplementary material for: Soil Bacterial Assemblage Across a Production Landscape: Agriculture Increases Diversity While Revegetation Recovers Community Composition
Source: Microb Ecol. 2023 Feb 10;85(3):1098–112. doi: 10.1007/s00248-023-02178-x (PMC10156840; doi:10.1007/s00248-023-02178-x)
Supplement: Supplementary file 1 — Supplementary file1 (DOCX 52 KB) [file 248_2023_2178_MOESM1_ESM.docx]

**Supplementary**

#### 1. Soil texture results

| Sample replicate ID | Landscape unit | % Clay | % Silt (2-20 micron) | % Sand (+20 micron) |
| --- | --- | --- | --- | --- |
| A1 | RemVeg | 9 | 9.9 | 81 |
| A2 | RemVeg | 13 | 25 | 62 |
| A3 | RemVeg | 12 | 21 | 67 |
| A4 | RemVeg | 8.8 | 12 | 79 |
| A5 | RemVeg | 11 | 12 | 77 |
| A6 | RemVeg | 12 | 14 | 74 |
| A7 | RemVeg | 12 | 22 | 66 |
| A8 | RemVeg | 14 | 26 | 60 |
| A9 | RemVeg | 14 | 18 | 68 |
| A10 | RemVeg | 11 | 15 | 74 |
| A11 | RemVeg | 9 | 4.8 | 86 |
| A12 | RemVeg | 12 | 14 | 74 |
| A13 | RemVeg | 9.5 | 11 | 80 |
| A14 | RemVeg | 32 | 21 | 47 |
| A15 | RemVeg | 24 | 25 | 51 |
| B1 | ExCrop | 17 | 31 | 52 |
| B2 | ExCrop | 12 | 23 | 65 |
| B3 | ExCrop | 13 | 24 | 63 |
| D1 | NewVineyard | 13 | 27 | 60 |
| D2 | NewVineyard | 14 | 22 | 64 |
| D3 | NewVineyard | 14 | 30 | 56 |
| D4 | NewVineyard | 12 | 22 | 66 |
| D5 | NewVineyard | 12 | 27 | 61 |
| G1 | Reveg | 11 | 8.4 | 81 |
| G2 | Reveg | 23 | 25 | 52 |
| G3 | Reveg | 22 | 25 | 53 |
| H1 | OldVineyard | 17 | 25 | 58 |
| H2 | OldVineyard | 18 | 24 | 58 |
| H3 | OldVineyard | 19 | 17 | 64 |
| H4 | OldVineyard | 19 | 24 | 56 |
| H5 | OldVineyard | 13 | 25 | 61 |
| H6 | OldVineyard | 13 | 25 | 62 |

#### 2. Alpha diversity under negative binomial GLM model

Significance codes: 0 ‘***’ 0.001 ‘**’ 0.01 ‘*’ 0.05 ‘.’ 0.1 ‘ ’ 1 (Holm-Bonferroni P-adjustment).

3. Alpha diversity pairwise comparisons under negative binomial GLM model

Significance codes: 0 ‘***’ 0.001 ‘**’ 0.01 ‘*’ 0.05 ‘.’ 0.1 ‘ ’ 1 (Holm-Bonferroni P-adjustment).

#### 4. Main and pairwise PERMANOVA on bacterial ASV communities

Significance codes Pr(> F): ‘ns’ not significant; ‘º’ P < 0.10; ‘*’ P < 0.05; ‘**’ P < 0.01; ‘***’ 880 P < 0.001.

#### 5. Observed plant functional diversity. “●” indicating plant functional group is present in landscape unit sample replicate.

| Landscape unit | Sample replicate | Annual groundcover | Perennial groundcover | Small Shrub | Medium/large shrub | Tree |
| --- | --- | --- | --- | --- | --- | --- |
| RemVeg | A1 | ● | ● | ● | ● | ● |
| RemVeg | A2 | ● |  | ● |  | ● |
| RemVeg | A3 | ● |  | ● |  | ● |
| RemVeg | A4 | ● |  | ● |  | ● |
| RemVeg | A5 | ● | ● | ● | ● | ● |
| RemVeg | A6 |  | ● | ● | ● | ● |
| RemVeg | A7 |  | ● | ● | ● | ● |
| RemVeg | A8 |  |  | ● |  | ● |
| RemVeg | A9 |  | ● | ● |  | ● |
| RemVeg | A10 |  |  | ● |  | ● |
| RemVeg | A11 |  | ● | ● |  | ● |
| RemVeg | A12 | ● | ● | ● | ● | ● |
| RemVeg | A13 | ● | ● | ● | ● | ● |
| RemVeg | A14 | ● | ● | ● | ● | ● |
| RemVeg | A15 |  | ● | ● |  | ● |
| Reveg | G1 | ● | ● |  | ● | ● |
| Reveg | G2 |  | ● |  | ● |  |
| Reveg | G3 | ● | ● |  |  |  |
| ExCrop | B1 | ● | ● | ● |  |  |
| ExCrop | B2 | ● | ● | ● |  |  |
| ExCrop | B3 | ● | ● |  |  |  |
| NewVineyard | D1 | ● |  |  | ● |  |
| NewVineyard | D2 | ● |  |  | ● |  |
| NewVineyard | D3 | ● |  |  |  |  |
| NewVineyard | D4 | ● |  |  | ● |  |
| NewVineyard | D5 | ● |  |  | ● |  |
| OldVineyard | H1 | ● |  |  | ● |  |
| OldVineyard | H2 | ● |  |  | ● |  |
| OldVineyard | H3 | ● |  |  | ● |  |
| OldVineyard | H4 | ● |  |  | ● |  |
| OldVineyard | H5 | ● |  |  | ● |  |
| OldVineyard | H6 | ● |  |  | ● |  |

6. Plant species list

Observed plant species across study site. “●” indicating plant species/genus/group is present in at least one sample replicate of the landscape unit.

| Plant Species | RemVeg | Reveg | ExCrop | OldVineyard | NewVineyard |
| --- | --- | --- | --- | --- | --- |
| Annual exotic grasses | ● | ● | ● | ● | ● |
| Perennial exotic grasses | ● | ● | ● | ● | ● |
| *Acacia melvillei* | ● |  |  |  |  |
| *Alectryon oleifolius* | ● | ● |  |  |  |
| *Austrostipa sp.* | ● | ● | ● |  |  |
| *Chenopod sp.* | ● | ● | ● |  |  |
| *Dodonaea viscosa* | ● |  |  |  |  |
| *Eremophila glabra* | ● |  |  |  |  |
| *Eremophila longifolia* | ● | ● |  |  |  |
| *Eucalyptus brachycalyx* | ● |  |  |  |  |
| *Eucalyptus gracilis* | ● |  |  |  |  |
| *Eucalyptus incrassata* | ● |  |  |  |  |
| *Eucalyptus leptophylla* | ● |  |  |  |  |
| *Exocarpus aphyllus* | ● |  |  |  |  |
| *Goodenia sp.* | ● |  |  |  |  |
| *Hakea leucoptera* | ● | ● |  |  |  |
| *Hakea tephrosperma* | ● |  |  |  |  |
| *Lomandra sp.* | ● |  |  |  |  |
| *Maireana sp.* | ● | ● | ● |  |  |
| *Myoporum platycarpum* | ● |  |  |  |  |
| *Olearia pimeleoides* | ● |  |  |  |  |
| *Pittosporum angustifolium* | ● |  |  |  |  |
| *Roepera apiculata* | ● |  |  |  |  |
| *Rytidosperma sp* |  |  | ● |  |  |
| *Senna artemisioides* | ● |  |  |  |  |
| *Tempeltonia rossii* | ● |  |  |  |  |
| *Teucrium racemosum* | ● |  |  |  |  |
| *Triticum sp.* |  | ● | ● |  |  |
| *Triodia scariosa* | ● |  |  |  |  |
| *Thysanotus tuberosus* | ● |  |  |  |  |
| *Vittadinia sp.* | ● | ● | ● |  |  |
| *Vitis sp.* |  |  |  | ● | ● |
| *Wahlenbergia sp.* |  |  |  |  |  |
| *Westringia rigida* | ● |  |  |  |  |

#### 7. Microbial inoculants applied to vineyard landscape units (OldVineyard and NewVineyard)

- Product A, comprised of; soil amendments designed to stimulate microbial activity (hydrolysed molasses, amino acids, fulvic acid, seaweed (Ascophyllum *nodosum* and *Durvillaea* species) and liquid fish; key nutrients (2.66% nitrogen w/v, 1.2% phosphorus w/v, .25% potassium w/v); and bacteria of family *Pseudomonadaceae* and of the group Actinomycetes. Applied at a rate of 10 L per hectare.
- Product B, comprised of; soil amendments (ash, fat, protein and carbohydrates), 2.09% phosphorus, bacteria of family *Pseudomonadacea* and of the group Actinomycetes. Applied at a rate of 10 L per hectare.

Products were applied via the irrigation system during peak growth periods, that being the austral spring/summer (September – Febuary).

| Bacterial family | RemVeg | OldVineyard | NewVineyard | ExCrop | Reveg |
| --- | --- | --- | --- | --- | --- |
| pooled (<2% relative abundance) | 33.2 | 37.6 | 35.1 | 30.8 | 38.7 |
| *Rubrobacteraceae* | 25.7 | 5.2 | 16.4 | 23.5 | 16.2 |
| *Bacillaceae* | 4.0 | 11.3 | 8.2 | 10.3 | 12.4 |
| *Bradyrhizobiaceae* | 7.5 | 5.5 | 6.2 | 7.5 | 9.3 |
| *Pseudonocardiaceae* | 7.0 | 2.6 | 2.6 | 3.1 | 2.8 |
| *Micrococcaceae* | * | 4.3 | 5.5 | 2.7 | 2.9 |
| *Geodermatophilaceae* | 2.4 | 2.8 | 3.0 | 2.8 | 2.5 |
| *Rhodospirillaceae* | 3.3 | 8.9 | 4.0 | 3.9 | 5.5 |
| *Beijerinckiaceae* | 2.8 | * | * | 2.4 | * |
| *Syntrophobacteraceae* | * | 2.8 | 2.5 | 2.8 | * |
| *Sphingomonadaceae* | 3.8 | 2.7 | 3.3 | 3.5 | 3.9 |
| *Sinobacteraceae* | 2.7 | 6.0 | 3.2 | 2.3 | * |
| *Hyphomicrobiaceae* | 3.3 | 2.8 | 2.0 | 2.3 | 3.1 |
| *Micromonosporaceae* | 2.1 | * | * | * | * |
| *Acetobacteraceae* | * | * | * | 2.2 | * |
| *Paenibacillaceae* | 2.3 | * | * | * | * |
| *Chitinophagaceae* | * | * | 2.4 | * | * |
| *Planococcaceae* | * | 2.8 | 2.9 | * | 2.6 |
| *Nocardioidaceae* | * | 2.1 | 2.6 | * | * |
| *Kouleothrixaceae* | * | 2.6 | * | * | * |

*Family under < 2% relative abundance in that landscape unit, included in pooled group.
